# Peripheral blood kinetics following total body irradiation and allogeneic hematopoietic stem cell transplantation: Timing matters

**DOI:** 10.1002/cam4.5452

**Published:** 2022-11-20

**Authors:** Cas S. Dejonckheere, Alexander M. C. Böhner, Eva Schmitz, Tobias A. W. Holderried, Leonard C. Schmeel, Peter Brossart, Frank A. Giordano, Mümtaz A. Köksal

**Affiliations:** ^1^ Department of Radiation Oncology University Hospital Bonn Bonn Germany; ^2^ Institute of Molecular Medicine and Experimental Immunology University Hospital Bonn Bonn Germany; ^3^ Department of Neurology University Hospital Bonn Bonn Germany; ^4^ Department of Oncology, Hematology, Immuno‐Oncology and Rheumatology University Hospital Bonn Bonn Germany; ^5^ Department of Radiation Oncology University Medical Center Mannheim Mannheim Germany

**Keywords:** acute lymphoblastic leukemia, acute myeloid leukemia, allogeneic hematopoietic stem cell transplantation, total body irradiation

## Abstract

Total body irradiation (TBI) remains an important component in many conditioning regimens before allogeneic hematopoietic stem cell transplantation (allo‐HSCT). Because of its frequent toxicity, patient selection is crucial, making it of interest to identify factors improving engraftment. In this retrospective single center analysis, the characteristics of 48 adult such patients were studied. Mean overall survival (OS) was 22.2 months after allo‐HSCT. Interestingly, people with an interval ≥3 days between TBI completion and allo‐HSCT showed improved OS, when compared to a shorter interval (*p* = 0.10). Peripheral blood kinetics after successful engraftment also differed, with a longer interval resulting in a higher platelet count and lower leukocyte and neutrophil (*p* < 0.05) count. These data suggest that the exact timing of TBI before allo‐HSCT might directly impact a patient's survival and could help single out those at higher risk of graft failure who might benefit from an altered conditioning regimen.

## Graphical abstract

Patients with longer interval between TBI completion and allo‐HSCT showed improved overall survival, when compared to a shorter interval. Exact timing of TBI before allo‐HSCT might directly impact a patient's survival and could help single out those at higher risk of graft failure who might benefit from an altered conditioning regimen.

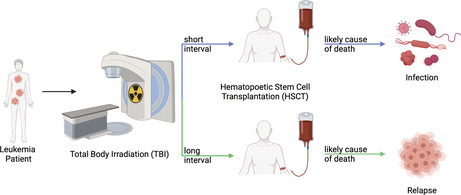

Total body irradiation (TBI) continues to play an important role in many conditioning regimens before allogeneic hematopoietic stem cell transplantation (allo‐HSCT).^1^, ^2^ Alongside chemotherapy, it serves a double purpose in patients with hematologic malignancies such as acute lymphoblastic or myeloid leukemia (ALL, AML): prevention of graft rejection by inducing immunosuppression and eradication of remaining malignant cells.^3^ Contrary to chemotherapy, radiation is able to better penetrate sanctuary organs (such as the brain and testes) since it is independent of blood supply and, furthermore, does not induce cross‐resistance with other agents.^1^, ^3^, ^4^ Accordingly, TBI‐containing regimens are a viable option for conditioning before allo‐HSCT in several hematologic malignancies.^5^, ^6^, ^7^, ^8^ Although efficacious and standard of care in many treatment protocols, TBI is still responsible for a plethora of side effects.^3^ Frequent acute toxicities includes nausea and vomiting, diarrhea, and pneumonitis, while late toxicities, such as hypothyroidism, cataracts, infertility, and secondary cancers are of particular concern, especially in the pediatric population receiving TBI.^9^, ^10^, ^11^ Standard regimens provide myeloablative total radiation doses up to 12 Gy, hyperfractionated into 2 × 2 Gy per day in order to reduce toxicity.^12^, ^13^ Additional dose escalation increases treatment‐related mortality (TRM), while a lower dose might result in graft failure and disease recurrence.^2^ In elderly patients or those with comorbidities limiting treatment options, non‐myeloablative chemoradiotherapy regimens are available with reduced radiation doses.^2^

It is of interest to identify any patient or treatment‐related factors leading to improved survival, as this could guide patient selection and facilitate treatment decisions, balancing survival benefit with TRM. This retrospective study aims to define such factors in a cohort of adult patients undergoing TBI.

We retrospectively analyzed the digital patient charts of all adult patients receiving TBI between January 2012 and January 2021 at our institution. The following data were extracted: age at TBI, sex, primary diagnosis, line of treatment, accompanying chemotherapy regimen, dose and fractionation of TBI, history of previous irradiation, time to allo‐HSCT, donor characteristics, hemoglobin, neutrophil, and platelet count at diagnosis and from day 1 until day 50 after allo‐HSCT, chimerism analysis (peripheral blood and bone marrow), transfusions, side effects and complications, and time to and cause of death. This study was approved by the local ethics committee (057/22). At our center, TBI is achieved using helical tomographic IMRT using a *Tomo Therapy* device (Accuray, Madison, Wisconsin, USA).

Kaplan–Meier curves were calculated to visualize overall survival (OS). Using the Mantel‐Cox logrank test, probabilities for differences between pairs of groups were extrapolated. For the statistical analysis of data comprised of two groups, unpaired Student's *t*‐tests were performed. In the case of three or more groups, simple one‐way ANOVA was utilized to detect statistical differences. A ROC analysis was performed to determine the optimal cutoff for the value of the interval between TBI completion and allo‐HSCT, generating two comparable groups. For the approximation of blood kinetics, exponential equations with plateau (four parameters) were calculated, beginning at the time of allo‐HSCT and reaching up to day 50 after transplantation. 95% confidence intervals are provided. A *p*‐value <0.05 was considered as statistically significant. GraphPad Prism (v.9.3.1) was used.

An overview of patient characteristics is outlined in Appendix S1. A total of 48 adult patients undergoing a 10/10 or 9/10 HLA‐matched allo‐HSCT were identified and included, with a median age of 49 years at the time of TBI (ranged from 18 to 70 years). ALL was the most common diagnosis (43.8%), followed by AML (25.0%). Three patients (6.3%) underwent an allo‐HSCT for the second time. One of these patients had already received TBI in the past. Disease status before allo‐HSCT was complete remission in 62.5% of cases and progressive disease in 14.6%. The administered radiation dose was 2 Gy, 4 Gy, 8 Gy, or 12 Gy in 10.4%, 16.7%, 43.8%, and 29.2% of cases, respectively. The median time between administration of the last TBI fraction and allo‐HSCT was 5 days (ranged from 0 to 7 days), and the main patient characteristics (age, primary diagnosis, TBI dose, HLA match) were comparable if the allo‐HSCT took place <3 days after TBI completion versus ≥3 days. A matched unrelated donor was used in 56.3%. TRM occurred in 40.7% of deceased patients at the time of assessment.

The evolution of hemoglobin, platelet, and leukocyte and neutrophil count until 50 days after allo‐HSCT is shown in Figure 1A–D. Patients receiving allo‐HSCT within 3 days after TBI completion yielded closely matching hemoglobin kinetics when compared to the subgroup in which allo‐HSCT was administered later (Figure 1A). For the platelets (Figure 1B), those receiving a later allo‐HSCT displayed significantly higher platelet counts compared to an earlier allo‐HSCT (*p* < 0.05). This divergence reached statistical significance around day 30 after allo‐HSCT. An inverted effect occurred for the overall leukocytes (Figure 1C) and neutrophils (Figure 1D), both reaching statistical significance around day 25 after allo‐HSCT.

**FIGURE 1:**
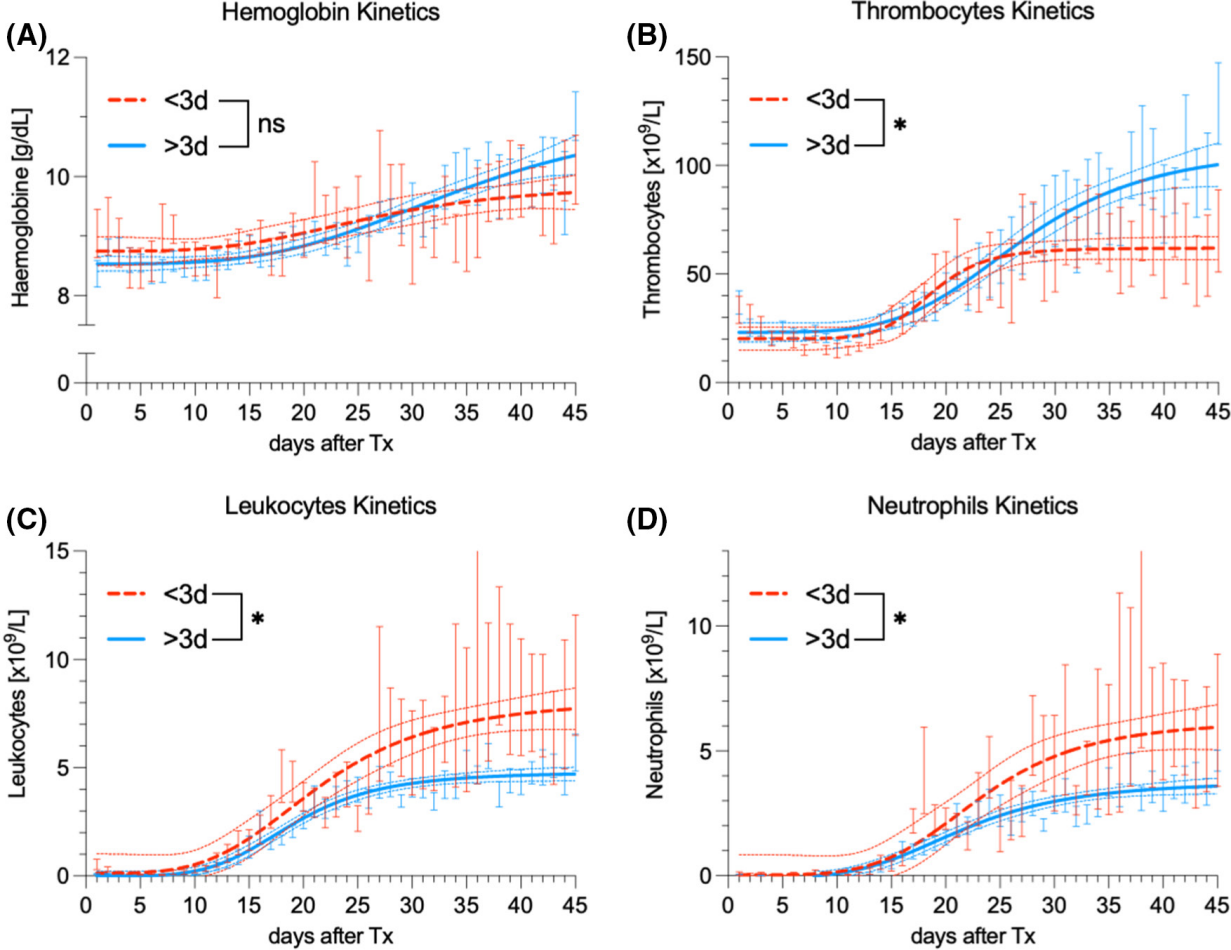
The evolution of (A) hemoglobin, (B) platelet, and (C) leukocyte and (D) neutrophil count until 50 days after allo‐HSCT as a nonlinear regression with plateau. The blue curve depicts an interval between TBI completion and allo‐HSCT of ≥3 days, the red curve <3 days. Non‐linear regression with 95% confidence intervals. *p < 0.05. allo‐HSCT, allogeneic hematopoietic stem cell transplantation; TBI, total body irradiation.

At the time of analysis, 21 patients (43.8%) were still alive. The mean OS in the first 36 months of follow‐up for this cohort was 22.2 months after allo‐HSCT. Relapse of the initial disease was the main cause of death, occurring in 16 patients (59.3%). Patients undergoing TBI and allo‐HSCT as a part of their primary treatment showed improved OS (*p* = 0.06; Figure 2A). High‐level chimerism in the peripheral blood and bone marrow was also associated with improved OS. Interestingly, people with an interval of ≥3 days between administration of the last TBI fraction and allo‐HSCT showed improved OS (*p* = 0.10; Figure 2B). The interval between TBI completion and allo‐HSCT displayed a mild positive correlation (*p* = 0.11; *R* ^2^ = 0.08) to the onset of acute graft‐versus‐host disease (Figure 2C). None of the other studied patient or treatment characteristics was predictive of engraftment success or improved OS.

**FIGURE 2:**
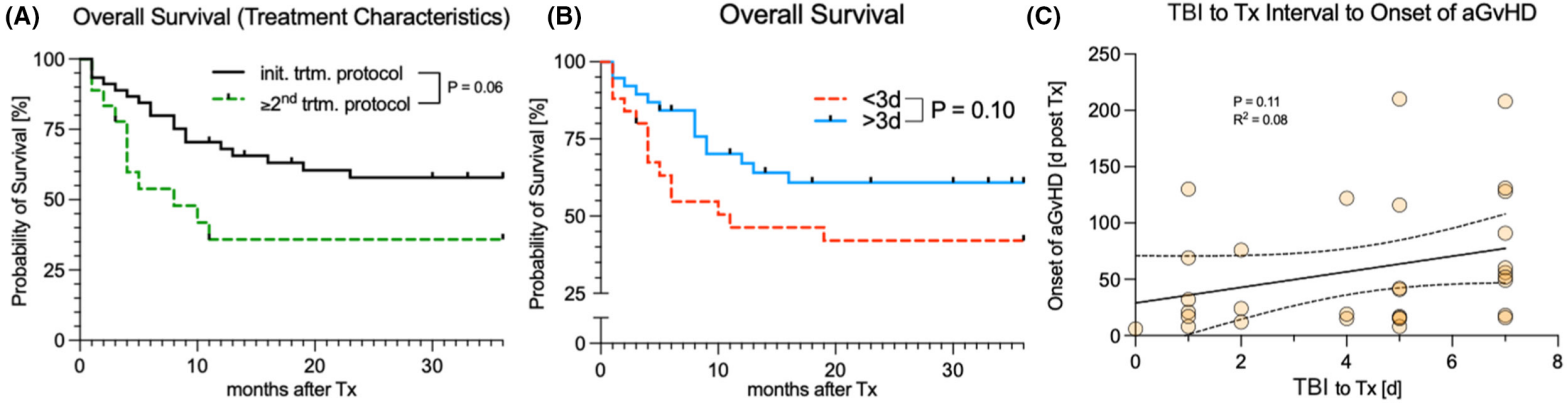
(A + B) Overall survival (OS) for the entire cohort. (A) Patients undergoing TBI and allo‐HSCT as a part of their primary treatment showed improved OS (*p* = 0.06). (B) Those with an interval of ≥3 days between administration of the last TBI fraction and allo‐HSCT showed improved OS (*p* = 0.10). (C) Mild positive correlation between this interval and the onset of acute graft‐versus‐host disease (*p* = 0.11; *R* ^2^ = 0.08). allo‐HSCT, allogeneic hematopoietic stem cell transplantation; TBI, total body irradiation.

In this retrospective single‐center analysis, we present a cohort of 48 adult patients who underwent TBI as a part of their conditioning regimen before 10/10 or 9/10 HLA‐matched allo‐HSCT for a malignant disorder.

Based on the peripheral blood kinetics (mainly of the neutrophils), we estimate that the engraftment in the entire group of patients took place between 15 and 20 days after allo‐HSCT, which is comparable to previous reports (Figure 1D).^14^

After successful engraftment, the platelet and leukocyte (including neutrophil) curves diverge, depending on the interval between TBI completion and allo‐HSCT. Regarding the neutrophil granulocytes, the subgroup with a shorter interval reaches a biologically effective level first, ranging around 7.0 × 10^9^ neutrophils/L by day 45 after allo‐HSCT, while the long‐interval subgroup reaches a mean value of 4.0 × 10^9^ neutrophils/L (Figure 1D). For platelets, however, the subgroup with the longer interval reaches values around 100 × 10^9^ platelets/L, with short‐interval subgroup coming short at 60 × 10^9^ platelets/L (*p* < 0.05; Figure 1B). Remarkably, platelets reach a plateau around day 25 after allo‐HSCT and do not increase from there on. The hemoglobin curves show no marked difference (Figure 1A).

Interestingly, a similar significant divergence is observed in the OS curves. In summary, this implicates that more time between TBI completion and allo‐HSCT results in a higher platelet count and lower leukocyte and neutrophil count. Accordingly, there is a positive correlation between this longer interval and OS, even though the risk of infection might be increased because of the lower leukocyte count.

A possible explanation might be sought within a differential repair window for malignant cells versus host cells (i.e., the tumor environment). On its turn, the planned interval between TBI completion and allo‐HSCT might guide clinicians in identifying patients in need of additional supportive measures (e.g., platelet transfusions, bone marrow stimulation, infection prophylaxis). Furthermore, the interval could be altered depending on individual patient and treatment characteristics, thus anticipating the most likely post‐transplant peripheral blood kinetics.

The degree of chimerism is predictive of graft failure and relapse, as was the case in this cohort.^15^ Other factors limiting the chances of successful engraftment in malignant disorders include ex vivo T‐cell depletion, HLA‐mismatched grafts, low transplanted donor cell doses, disease status other than CR, or underlying disease.^16^, ^17^, ^18^, ^19^

This study harbors several limitations, most notably its retrospective nature and relatively small sample size compiled of a heterogeneous study population (primary diagnosis, disease status before allo‐HSCT, administered TBI dose, donor characteristics) from a single‐center, with partially incomplete data on some important aspects (e.g., conditioning intensity or minimal residual disease status in the subgroup with acute leukemia; a known significant predictive and prognostic factor).^20^ The small number of patients in their respective subgroups did not make an accurate stratification possible in the current cohort. Hematologic malignancies span a wide spectrum and can occur at all ages, rendering the treatment protocols very diverse. Furthermore, the timing of allo‐HSCT after TBI completion is often based on historical evidence. Additionally, peripheral blood kinetics are influenced by numerous factors. In hematologic disorders in particular, patient characteristics, disease, and treatment play an important role and can directly influence the outcome. The (molecular) factors behind these findings also require further investigation. We do, however, reckon that the current study provides a relevant overview of a cohort of adult TBI‐treated patients from a single comprehensive cancer center. Larger prospective trials are needed to further elucidate the exact impact of the interval between TBI completion and allo‐HSCT and its impact on engraftment and outcome, with stratification according to primary diagnosis, disease status, and conditioning intensity.

In the modern era of allo‐HSCT, graft failure remains a complex obstacle, often with a multifactorial etiology. Even with improved monitoring and supportive therapy in recent years, it continues to hold a poor prognosis.^16^, ^17^, ^21^, ^22^ Although it has been proposed that conditioning regimens with (high dose) TBI reduce the risk of graft failure, comparative studies are lacking.^21^ Large, prospective trials with homogenous patient collectives are needed, in order to unequivocally establish patient and treatment‐related factors predictive of successful engraftment. The current data suggest that the exact timing of allo‐HSCT after TBI might directly impact a patient's survival. If this finding is replicated, it could guide patient and protocol selection and single out those at higher risk of graft failure and potential death, thus identifying those who might benefit from an altered conditioning regimen or additional supportive measures. Until then, a multidisciplinary approach regarding TBI dose and timing in allo‐HSCT is required, in order to optimize patient care and outcome.

## AUTHOR CONTRIBUTIONS

**Cas S Dejonckheere:** Formal analysis (equal); validation (equal); writing – original draft (lead); writing – review and editing (equal). **Alexander M.C. Böhner:** Formal analysis (equal); validation (equal); visualization (lead); writing – review and editing (equal). **Eva Schmitz:** Conceptualization (equal); data curation (lead); investigation (equal); validation (equal). **Tobias A.W. Holderried:** Formal analysis (equal); supervision (equal); validation (equal); writing – review and editing (equal). **Christopher L Schmeel:** Formal analysis (supporting); validation (supporting); writing – review and editing (supporting). **Peter Brossart:** Formal analysis (supporting); validation (supporting); writing – review and editing (supporting). **Frank A Giordano:** Formal analysis (supporting); validation (supporting); writing – review and editing (supporting). **Mümtaz A. Köksal:** Conceptualization (lead); data curation (supporting); formal analysis (supporting); methodology (lead); project administration (lead); supervision (lead); validation (equal); writing – review and editing (supporting).

## FUNDING INFORMATION

This research received no external funding.

## CONFLICT OF INTEREST

The authors declare no conflict of interest.

## Supporting information


Appendix S1

## Data Availability

The data that support the findings of this study are available on request from the corresponding author, MK. The data are not publicly available due to their containing information that could compromise the privacy of research participants.
