# Supplementary material for: Peripheral blood kinetics following total body irradiation and allogeneic hematopoietic stem cell transplantation: Timing matters
Source: Cancer Med. 2022 Nov 20;12(6):7170–4. doi: 10.1002/cam4.5452 (PMC10067066; doi:10.1002/cam4.5452)
Supplement: Supplementary file 1 — Appendix S1 [file CAM4-12-7170-s001.docx]

**SUPPORTING INFORMATION/SUPPLEMENT**

**Supplement A**. Graphical abstract.


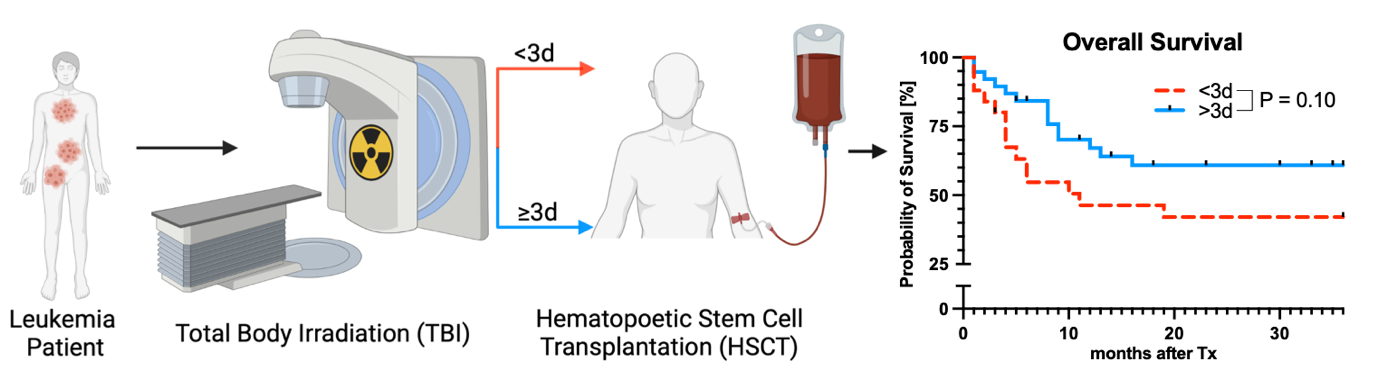


**Supplement B**. Overview of patient characteristics (*n* = 48).

AML = acute myeloid leukemia; ALL = acute lymphoblastic leukemia; allo-HSCT = allogeneic hematopoietic stem cell transplantation; TBI = total body irradiation; CR = complete remission; PD = progressive disease; Flu = fludarabine; Eto = etoposide; Cyclo = cyclophosphamide; Mel = melphalan; Cy = cytarabine; MRD = matched related donor; MUD = matched unrelated donor; MMUD = mismatched unrelated donor; HLA = human leukocyte antigen; SD = standard deviation; RRM = relapse-related mortality; TRM = treatment-related mortality.

| Median age (range) in years | | | | 49 (18−70) | |
| --- | --- | --- | --- | --- | --- |
|  | | < 3 d | | | 55 ± 11.5 |
|  | | ≥ 3 d | | | 43 ± 16.9 |
| Sex | | |  | ***n* (%)** | |
|  | male | | | 22 (45.8) | |
|  | female | | | 26 (54.2) | |
| Diagnosis | | |  |  | |
|  | ALL | | | 21 (43.8) | |
|  | | < 3 d | | | 6 (40) |
|  | | ≥ 3 d | | | 15 (44.1) |
|  | AML | | | 12 (25.0) | |
|  | | < 3 d | | | 2 (12.5) |
|  | | ≥ 3 d | | | 10 (29.4) |
|  | lymphoma | | | 4 (8.3) | |
|  | | < 3 d | | | 1 (6.3) |
|  | | ≥ 3 d | | | 3 (8.8) |
|  | other | | | 11 (22.9) | |
|  | | < 3 d | | | 7 (43.8) |
|  | | ≥ 3 d | | | 4 (11.8) |
| Treatment characteristics | | |  |  | |
|  | previous allo-HSCT | | | 3 (6.3) | |
|  | previous TBI | | | 1 (2.1) | |
| Disease status | | |  |  | |
|  | CR | | | 30 (62.5) | |
|  | PD | | | 7 (14.6) | |
|  | other | | | 11 (22.9) | |
| Administered TBI dose | | |  |  | |
|  | 2 Gy | | | 5 (10.4) | |
|  | | < 3 d | | | 5 (50.0) |
|  | | ≥ 3 d | | | 0 (0.0) |
|  | 4 Gy | | | 8 (16.7) | |
|  | | < 3 d | | | 2 (20.0) |
|  | | ≥ 3 d | | | 6 (15.8) |
|  | 8 Gy | | | 21 (43.8) | |
|  | | < 3 d | | | 2 (20.0) |
|  | | ≥ 3 d | | | 19 (50.0) |
|  | 12 Gy | | | 14 (29.2) | |
|  | | < 3 d | | | 1 (10.0) |
|  | | ≥ 3 d | | | 13 (34.2) |
| Administered chemotherapy (main component) | | | | | |
|  | Flu | | | 26 (54.2) | |
|  | | < 3 d | | | 6 (60.0) |
|  | | ≥ 3 d | | | 20 (52.6) |
|  | Eto | | | 5 (10.4) | |
|  | | < 3 d | | | 0 (0.0) |
|  | | ≥ 3 d | | | 5 (13.2) |
|  | Cyclo | | | 8 (16.7) | |
|  | | < 3 d | | | 1 (10.0) |
|  | | ≥ 3 d | | | 7 (18.4) |
|  | Flu + Cyclo | | | 3 (6.3) | |
|  | | < 3 d | | | 2 (20.0) |
|  | | ≥ 3 d | | | 1 (2.6) |
|  | Flu + Mel | | | 2 (4.2) | |
|  | | < 3 d | | | 0 (0.0) |
|  | | ≥ 3 d | | | 2 (5.3) |
|  | Flu + Cyclo + Cy | | | 4 (8.3) | |
|  | | < 3 d | | | 1 (10.0) |
|  | | ≥ 3 d | | | 3 (7.9) |
| Donor | | |  |  | |
|  | MRD | | | 11 (22.9) | |
|  | MUD | | | 27 (56.3) | |
|  | MMUD | | | 10 (20.8) | |
| HLA-match | | |  |  | |
|  | 10/10 | | | 38 (79.2) | |
|  | | < 3 d | | | 10 (100.0) |
|  | | ≥ 3 d | | | 28 (73.7) |
|  | 9/10 | | | 10 (20.8) | |
|  | | < 3 d | | | 0 (0.0) |
|  | | ≥ 3 d | | | 10 (26.3) |
| Graft source | | | | | |
|  | peripheral blood | | | 38 (79.2) | |
|  | bone marrow | | | 10 (20.8) | |
| Median stem cell dose ± SD | | | | 5.79 ± 2.46 × 10^6^/kg | |
| Main cause of death | | |  |  | |
|  | RRM | | | 16 (59.3) | |
|  | TRM | | | 11 (40.7) | |
